# Design of a Metal-Oxide Solid Solution for Sub-ppm H_2_ Detection

**DOI:** 10.1021/acssensors.1c02481

**Published:** 2022-02-16

**Authors:** Elena Spagnoli, Andrea Gaiardo, Barbara Fabbri, Matteo Valt, Soufiane Krik, Matteo Ardit, Giuseppe Cruciani, Michele Della Ciana, Lia Vanzetti, Gabriele Vola, Sandro Gherardi, Pierluigi Bellutti, Cesare Malagù, Vincenzo Guidi

**Affiliations:** † Department of Physics and Earth Sciences, University of Ferrara, via Giuseppe Saragat 1, Ferrara 44122, Italy; ‡ MNF-Micro Nano Facility Sensors and Devices Center, Bruno Kessler Foundation, via Sommarive 18, Trento 38123, Italy; § Sensing Technologies Lab, Faculty of Science and Technology, Free University of Bozen-Bolzano, piazza Università 1, Bolzano 39100, Italy; ∥ National Research Council, Institute for Microelectronics and Microsystems, via Gobetti 101, Bologna 40129, Italy; ⊥ Cimprogetti S.r.l. Lime Technologies, via Pasubio, Bergamo 24044, Italy

**Keywords:** (Sn,Ti,Nb)*_x_*O_2_, metal-oxide solid solution, H_2_ detection, chemoresistive gas sensors, nanostructured MOX

## Abstract

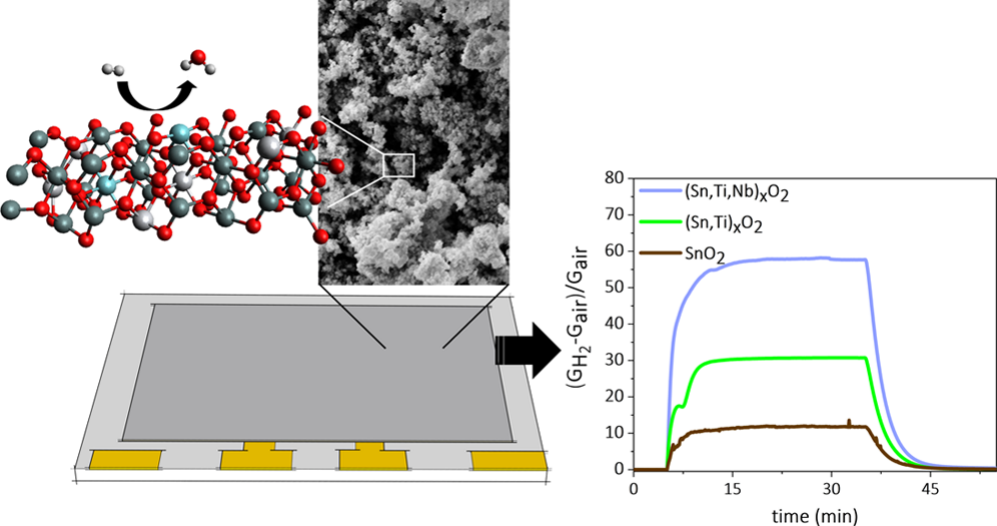

Hydrogen is largely adopted in industrial processes and is one of the leading options for storing renewable energy. Due to its high explosivity, detection of H_2_ has become essential for safety in industries, storage, and transportation. This work aims to design a sensing film for high-sensitivity H_2_ detection. Chemoresistive gas sensors have extensively been studied for H_2_ monitoring due to their good sensitivity and low cost. However, further research and development are still needed for a reliable H_2_ detection at sub-ppm concentrations. Metal-oxide solid solutions represent a valuable approach for tuning the sensing properties by modifying their composition, morphology, and structure. The work started from a solid solution of Sn and Ti oxides, which is known to exhibit high sensitivity toward H_2_. Such a solid solution was empowered by the addition of Nb, which—according to earlier studies on titania films—was expected to inhibit grain growth at high temperatures, to reduce the film resistance and to impact the sensor selectivity and sensitivity. Powders were synthesized through the sol–gel technique by keeping the Sn–Ti ratio constant at the optimal value for H_2_ detection with different Nb concentrations (1.5–5 atom %). Such solid solutions were thermally treated at 650 and 850 °C. The sensor based on the solid solution calcined at 650 °C and with the lowest content of Nb exhibited an extremely high sensitivity toward H_2_, paving the way for H_2_ ppb detection. For comparison, the response to 50 ppm of H_2_ was increased 6 times vs SnO_2_ and twice that of (Sn,Ti)*_x_*O_2_.

The current scenario of industrial development combined with a dramatic population growth results in a high demand for energy. Thereby, scientific and technological activities have been focused on the development of sustainable energy sources, aimed at reducing the carbon footprint of energy production processes.^1^ Within this framework, hydrogen-based energy generation has become a globally accepted practice to obtain clean energy.^2^ Moreover, hydrogen (H_2_) is largely employed in various industrial processes, such as methanol and ammonia production and petroleum refinery. In 2019, the global market of hydrogen generation was valued at USD 117.49 billion, and it is expected to grow steadily in the future.^3^ However, the production, storage, and transportation of H_2_ are very risky because of its high energy density and explosivity even at concentrations as low as 4%,^2^ and therefore, H_2_ monitoring is a relevant challenge.

Several technologies have been developed to monitor H_2_ concentration. Analytical techniques, such as quadrupole mass spectrometry, gas chromatography, mid-infrared spectroscopy, atomic absorption spectrometry, and proton-transfer reaction mass spectrometry, can reliably detect H_2_ even in traces.^4−8^ However, instruments large size and weight, time-consuming process, high cost, lack of portability, need for continuous maintenance, and calibration, along with the requirement of trained personnel,^2^ have negatively impacted the widespread use of such analytical tools in many applications.

The advent of solid-state gas sensors, such as chemoresistive gas sensors, has partially overcome these issues, and several types of small and low-cost gas sensors have been investigated for H_2_ detection.^2^

About 90% of the metal oxides (MOXs) utilized as sensing films in chemoresistive devices belong to a restricted class of semiconductors, i.e., SnO_2_, ZnO, TiO_2_, WO_3_, In_2_O_3_, and CuO.^9^ The paucity of reactive metal oxides may limit the sensing performance expressed by the 3S rule (selectivity, sensitivity, and stability).^10,11^ A solid solution of two individually sensitive metal oxides would allow to vary with continuity the sensing properties of the solution, with the relative proportion of the metal as a free parameter to be optimized. The aim is to magnify the qualities of single oxides and to possibly mitigate their deficiencies. As a practical example, a solid solution of Sn and Ti oxides combines high sensitivity toward reducing gases typical of SnO_2_^12−17^ and the peculiar low influence by humidity on the sensing properties of TiO_2_.^12,16^

Addition of a metal to a sensing metal oxide or to a sensing solid solution may also be beneficial.^18−21^ Ferroni and Guidi et al. first investigated the effect of Nb^5+^ incorporation within a TiO_2_ lattice, proving that it can inhibit grain growth and anatase-to-rutile transition.^22,23^ Moreover, Nb doping increased the bulk conductivity of titania.^24^ Carotta et al. demonstrated the influence of Nb on the solid solution of Sn and Ti for a single level of concentration of Nb.^25^

The aim of this article is to study H_2_ sensing by varying Nb concentration in a solid solution of Sn and Ti oxides. Such a material was obtained through the sol–gel technique using different Nb concentrations (1.5 to 5 atom %), while the Sn–Ti ratio was kept constant to 70:30 mol/mol, i.e., the best Sn–Ti proportion for H_2_ detection.^25^ Such solid solutions were thermally treated at 650 and 850 °C. The sensor based on the solid solution calcined at 650 °C and with the lowest content of Nb exhibited an extremely high sensitivity toward H_2_ and significant selectivity. The films exhibited relevant responses even down to 0.4 ppm with an extraordinary potential to sense through almost the whole ppb range. The effect of possible interferents for applications, e.g., carbon monoxide, methane, nitrogen dioxide, ethanol, and acetaldehyde, resulted in a marginal influence on the response.

## Experimental Section

### Synthesis of SnO_2_, (Sn,Ti)*_x_*O_2_, and (Sn,Ti,Nb)*_x_*O_2_ Powders

All of the chemicals, with reagent-grade purity and without any further purification, were from Sigma-Aldrich. Double-distilled water, passed through a Millipore Elix water purification system, was used.

SnO_2_ and (Sn,Ti)*_x_*O_2_ powders were prepared according to the procedures previously described.^16,26^ Both powders were calcined at 650 °C for 2 h under air in a muffle oven.

Different samples of (Sn,Ti,Nb)*_x_*O_2_ were prepared by maintaining a Sn–Ti molar ratio of 70:30 and modifying the content of Nb to 1.5 and 5 atom %. In a typical synthesis procedure, required stoichiometric proportions of Sn(II)ethylhexanoate, Ti(IV)butoxide, and NbCl_5_ were dissolved in a hydroalcoholic solution. The total cation concentration was kept at [M^*n*+^]_tot_ = 0.1 M for each synthesis batch. The three-component (Sn,Ti,Nb) solution was obtained by dissolving Ti(IV)butoxide and NbCl_5_ separately in two different beakers each containing 35 mL of 2-propanol. The two transparent solutions were mixed in an ice bath, and Sn(II)ethylhexanoate was slowly added. Then, 1 mL of 0.1 M HNO_3_ solution was added dropwise to hydrolyze the metal–organic molecules, and 30 mL of mQ water was added to achieve a pale yellow colloid. The entire process was carried out by maintaining the solution under gentle stirring. The colloidal solution was kept in the ice bath for 3 h and left to rest overnight. The colloid was separated from the solution by vacuum filtration and washed several times with 2-propanol and water. The resultant xerogel was dried in air at 110 °C for 4h and calcined at either 650 or 850 °C for 2 h under air in a muffle oven.

As summarized in Table 1, SnO_2_, (Sn,Ti)*_x_*O_2_, and (Sn,Ti,Nb)*_x_*O_2_ powders were labeled according to the molar ratio between Sn, Ti, and Nb used for the synthesis and the calcination temperature.

**Table 1:** SnO_2_, (Sn,Ti)*_x_*O_2_, and (Sn,Ti,Nb)*_x_*O_2_ Sample Label According to the Molar Ratio between Sn, Ti, and Nb Used for the Synthesis and the Calcination Temperature

Sn/Ti/Nb molar ratio	calcination temperature (°C)	label
100	650	SnO_2_ 650
70:30:0	650	ST30 650
66.5:28.5:5	650	STN 5 650
66.5:28.5:5	850	STN 5 850
69.0:29.5:1.5	650	STN 1.5 650
69.0:29.5:1.5	850	STN 1.5 850

### Material Characterization

The chemical composition and the morphology of the synthesized powders were investigated by energy-dispersive X-ray spectroscopy (EDX) and scanning electron microscopy (SEM), using a Zeiss EVO 40 microscope. Gas porosity and specific surface area of the powders were explored using a Micromeritics TriStar II Plus automated gas sorptometer. X-ray powder diffraction (XRPD) data were collected at room temperature on a Bruker D8 Advance Da Vinci diffractometer. X-ray photoelectron spectroscopy (XPS) analyses were performed using a Kratos AXIS Ultra DLD. XPS quantification was performed using the instrument sensitivity factors and high-resolution scans.

All specifications about instruments, equipment, data collection, and evaluation are reported in the Supporting Information.

### Gas Sensor Preparation and Characterization

The powders were ground in an agate mortar and mixed with organic vehicles to form homogeneous pastes. Then, the pastes were screen-printed onto alumina substrates, equipped with interdigitated gold electrodes on the top side and a heater on the back side.^27,28^ The deposition thickness for each device was about 20–30 μm, with an area of 1.22 × 1.60 mm^2^.^27^ The devices were calcined at 650 °C for 2 h and finally packaged with a standard TO-39 support using thermocompression bonding and a gold wire.^27,29^

The sensing properties of STN sensors were investigated using a dedicated apparatus, including a customized sealed gas test chamber and a data acquisition system.^30^ All specifications about the system for gas injection, chamber characteristics, and electronics are given in the Supporting Information.

The sensor response was defined as
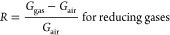
1
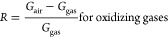
2where *G*_air_ and *G*_gas_ are the steady-state conductance values in air and gas, respectively.

The gas sensing characterization was carried out under both dry (20% of O_2_ and 80% of N_2_) and wet conditions.

Sensor signal baselines were stabilized at the beginning of each measurement by keeping the sensors at their working temperature under a continuous flow (500 sccm) of either synthetic dry or wet air, in the range of 1.7–55% relative humidity (RH %).

The response and recovery times were calculated as the time needed to reach 90% of the response value and the time required to switch back to 90% of the baseline value, respectively.

To estimate the best working temperature, the responses to 50 ppm of H_2_ in dry air were compared by heating the film to four different temperatures (350, 400, 450, and 500 °C). The sensitivity was examined by exposing the sensors to increasing H_2_ concentrations. The repeatability of the dynamic responses vs 0.4 and 100 ppm of H_2_, i.e., the extreme concentrations of the calibration curves, was studied for the sensors with better sensitivity.

To investigate the sensor selectivity vs H_2_, the films were exposed to water vapor and several possible interfering gases such as carbon monoxide (CO), methane (CH_4_), acetaldehyde (C_2_H_4_O), ethanol (C_2_H_5_OH), and nitrogen dioxide (NO_2_). For the sake of comparison, all of the concentrations were set at the same level as that of H_2_, i.e., 10 ppm. The only exception was NO_2_, which was set at 1 ppm, which is its low TLV (threshold limit value) for short-term exposure based on European directives.^31^

The investigation of selectivity through CO and CH_4_ is mandatory because they are both involved in steam reforming of methane (SRM), i.e., the predominant industrial process for manufacturing hydrogen and syngas. Here, steam endothermically reacts with methane to produce H_2_ and CO.^32,33^ Moreover, the films were probed vs an aldehyde (C_2_H_4_O), an alcohol (C_2_H_5_OH), and an oxidizing gas (NO_2_), which are thought as representative of chemical compounds with different functional groups.

The Arrhenius plots and energy barrier measurements in air and vs 25 ppm H_2_ were carried out for all of the films.^34^

## Results

### Morphological, Structural, and Chemical Characterization

**Figure 1:**
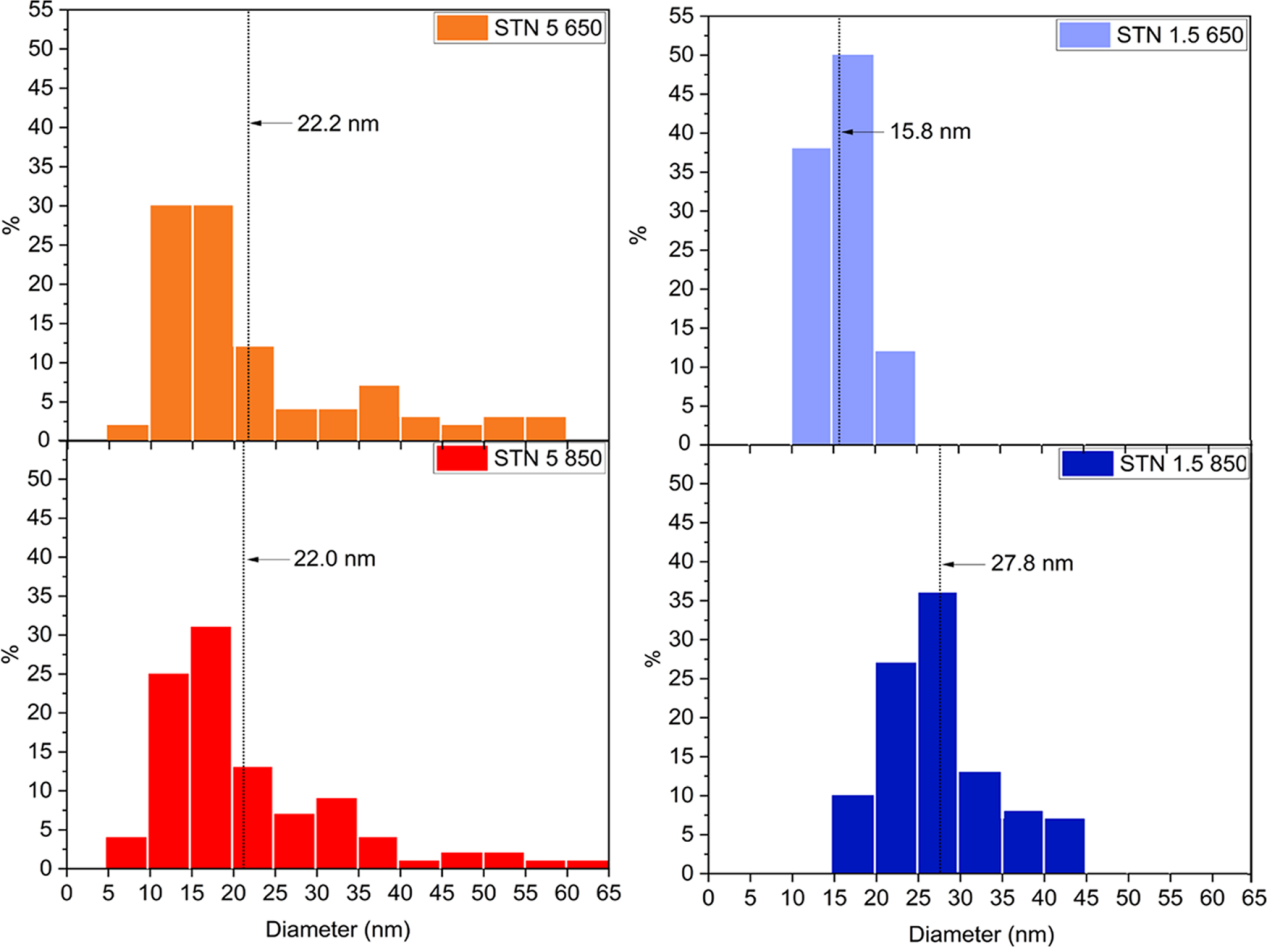
Distribution of particle diameters (nm) in STN samples, revealing the influence of annealing temperature on grain coalescence. Black dotted lines indicate the mean value of the distributions.

Powder morphologies were observed by SEM, as shown in Figure S1. All of the samples were composed of spheroidal nanograins. Figure 1 shows the particle-size distribution of the powders with Nb contents of 1.5 and 5 atom % and at calcination temperatures of 650 and 850 °C. Particle sizes of STN 1.5 650 ranged within 10–15 nm, while a wider distribution ranging within 25–60 nm was recorded for STN 5 650. The relative proportion of Sn, Ti, and Nb played a fundamental role in particle growth in solution since a higher concentration of Nb^5+^ led to a wider dispersion of the particle-size distribution. At the same time, the addition of a higher concentration of Nb^5+^ prevented grain coalescence at high temperatures.^23^ Indeed, for the sample STN 5 850, the distribution of the particle diameters remained almost unchanged compared to STN 5 650. On the contrary, the average particle size increased from 15.8 nm at 650 °C to about 27.8 nm at an 850 °C firing temperature for the STN 1.5 samples.

To further investigate the influence of temperature on sample morphology, the adsorption and desorption isotherms of representative samples were carried out (Figure S2).

The corresponding N_2_-Brunauer–Emmett–Teller (BET) surface area and the average pore size diameters are reported in Table 2. The BET specific surface area of the powders treated at 650 °C was similar. The treatment at 850 °C of the powder with 1.5 atom % of Nb halved its BET specific surface area compared to its counterpart when heated at 650 °C. From the calculated pore size, it can be inferred that the sample exhibited mesoporosity.^35^ The samples heated at 650 °C showed an average pore diameter remarkably lower than that of STN 1.5 850.

**Table 2:** BET Specific Surface Areas and Pore Size Distributions of Representative Solid Solution Powders, Highlighting Dissimilarity Especially between Samples with Different Heat Treatments

sample	specific surface area by BET method (m^2^/g)	pore size: D-H desorption average pore diameter (4V/A) (nm)
ST30 650	50.42	12.66
STN 1.5 650	43.72	16.71
STN 5 650	48.36	12.52
STN 1.5 850	22.63	23.95

**Figure 2:**
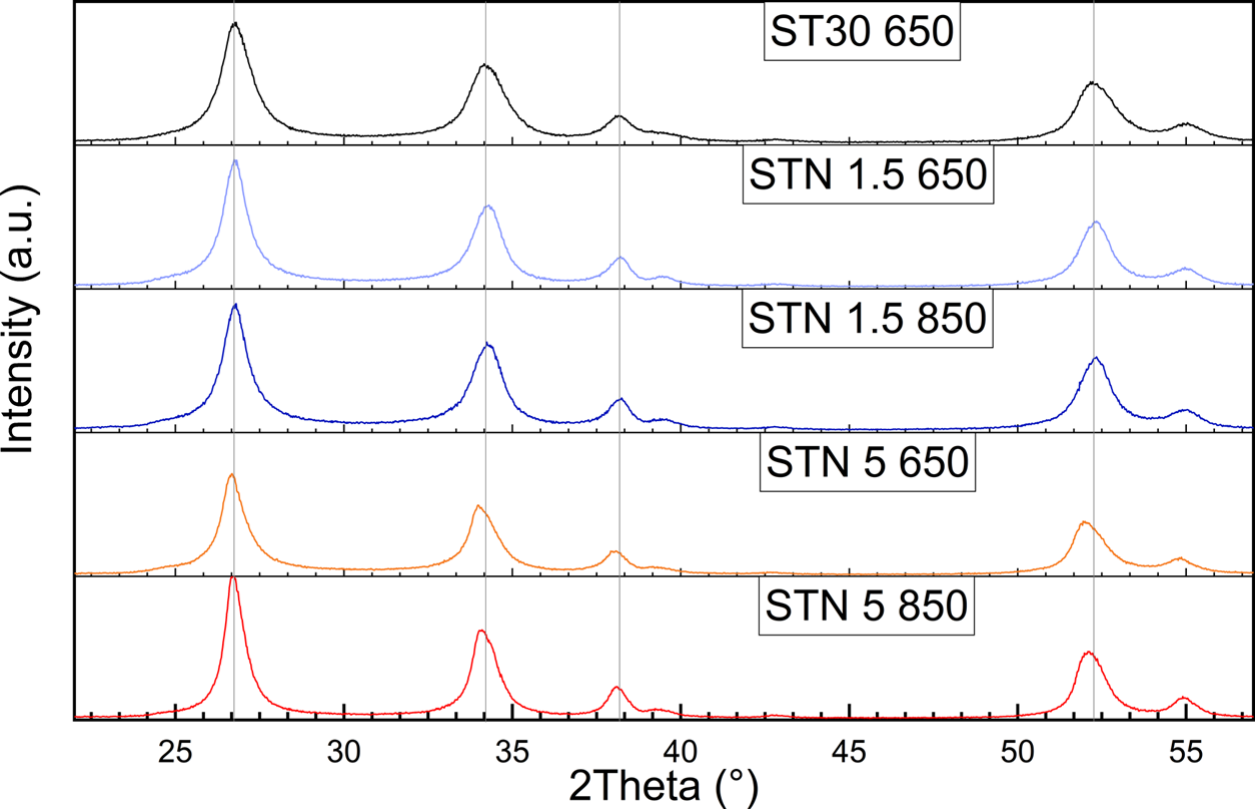
X-ray powder diffraction patterns collected at RT. Gray lines parallel to the *y* axis mark peak positions for ST30 650 to highlight the shift for STN peaks. The fractions of rutile-type (s.g. *P*4_2_/*mnm*) and anatase-type (s.g. *I*4_1_/*amd*) phases were ∼98 and 2 wt %, respectively.

XRPD patterns collected at room temperature in the 2θ angular range of 22–57° are shown in Figure 2. Peak positions for ST30 650 were shifted toward higher 2θ angles compared to those for STN samples as a result of smaller lattice parameters. In all of the investigated samples, the main phase (ranging from 96.9 to 99.0 wt %, see Table S1) was a tetragonal rutile-type phase (space group, s.g. *P*4_2_/*mnm*). A variable amount of tetragonal anatase-type phase (s.g. **I**4_1_/*amd*) was detected as the remaining fraction. The main rutile-type phase (starting structural model from Hirata)^36^ along with the associated anatase-type phase (starting structure model from Howard et al.)^37^ was modeled by carrying out a multiphase refinement in which only the scale factors and unit-cell parameters were varied. As an example of the approach used for the XRPD pattern analysis, the Rietveld refinement plot for the sample STN 1.5 650 in the 2θ range of 10–90° is shown in Figure S3.

Useful information on the synthesized solid solution can be achieved by plotting the lattice parameter ratio (*a*/*c*) of the rutile-type phase versus its volume (*V*) (Figure S4). By contrasting the lattice parameters of the investigated samples to those of pure TiO_2_ and SnO_2_ from the literature, it emerged that the rutile-type phase was a solid solution where the smaller ^[6]^Ti^4+^ (ionic radius, i.r. = 0.605 Å) and ^[6]^Nb^5+^ (i.r. = 0.64 Å) replace about 17% of the larger ^[6]^Sn^4+^ (i.r. = 0.69 Å) at the octahedral site (ionic radii from Shannon).^38^ Nb incorporation in the solid solution of Sn and Ti resulted in a bigger cell volume (Table S1), especially for samples STN 5 650 and 850. On the other hand, the unit-cell volume of the anatase-type phase was close to that of other TiO_2_ nano-anatase from the literature.

EDX and XPS characterizations allowed us to estimate the composition of the films in the bulk and at the surface (see Table 3). The Sn/Ti/Nb atomic proportion obtained through EDX was about 72:23:5 and 74:24.4:1.6 for STN 5 and STN 1.5, respectively. The obtained atomic proportion was slightly different from the synthesis starting stoichiometry, and a relative deficiency of Ti was observed. XRPD phase analysis revealed the presence of a small amount of nano-anatase phase mainly composed of TiO_2_. Such small nanoparticles can be partly lost during the supernatant filtration and powder washing, explaining the relative deficiency of Ti in the final samples compared to that in the batch of synthesis. The proportion of Sn, Ti, and Nb obtained through the XPS analysis revealed higher contents of Ti and Nb than those detected by the EDX analysis (Table 3), an evidence that Ti and Nb partially segregated on the surface of the STN nanoparticles. The same behavior has been already observed in the synthesis of other MOX solid solutions and perovskites, in which the driving force for a surface segregation was the lower surface energy of the component that segregated at the surface, as well as a difference in cation mobility under an applied gradient.^39,40^

**Table 3:** Compositional Proportion of Sn, Ti, and Nb in STN Samples, Obtained through EDX and XPS analyses, Highlighting the Slightly Different Composition of Bulk and Surface

EDX	STN 1.5 650	STN 5 650	STN 1.5 850	STN 5 850
Ti	22.0	22.7	26.7	21.2
Nb	1.7	5.3	1.4	5.0
Sn	76.3	72.0	71.9	73.8

XPS	STN 1.5 650	STN 5 650	STN 1.5 850	STN 5 850
Ti	29.3	30.2	29.4	25.2
Nb	3.8	9.8	3.4	12.4
Sn	66.9	60.0	67.2	62.4

The differences in Sn, Ti, and Nb composition between STN 1.5 and 5 evidenced by EDX and XPS analyses would impact the bulk and surface properties of the materials, influencing their behaviors as a gas sensor.

Figure S5 shows the high-resolution scans of Sn 3d, Ti 2p, and Nb 3d core levels. The high-resolution spectra of the Sn 3d and Nb 3d regions showed peaks located at binding energies Sn 3d_3/2_ = 594.9 eV, Sn 3d_5/2_ = 486.5 eV and Nb 3d_3/2_ = 207.1 eV, Nb 3d_5/2_ = 209.7 eV, ascribed to Sn^4+^ and Nb^5+^, respectively.^41,42^ The peaks in the high-resolution spectrum of the Ti 2p region, mostly attributed to the Ti^4+^ oxidation state at binding energies Ti 2p_1/2_ = 464.5 eV and Ti 2p_3/2_ = 458.7 eV,^43^ showed a shoulder due to a small content of Ti^3+^ (Ti 2p_1/2_ = 463.3 eV and Ti 2p_3/2_ = 457.0 eV).^43,44^ Sn 3d, Ti 2p, and Nb 3d core-level fits for STN 5 850 are displayed as an example in Figure S6.

### Gas Sensing Properties

The optimal response of STN films was determined by measuring the conductance change before and after injection of 50 ppm of H_2_ at different working temperatures within 350–500 °C. Two different trends were observed (Figure S7). The influence of the working temperature on the response of STN sensors calcined at 650 °C was significant. Indeed, at 450 °C, the response peaked 2.5 times more than that recorded at 400 and 500 °C. On the other hand, the responses of the STN sensors calcined at 850 °C moderately increased with working temperature. Hereinafter, the working temperature was set at 450 °C for all STN sensors to compare their sensing properties under the same operating condition.

**Figure 3:**
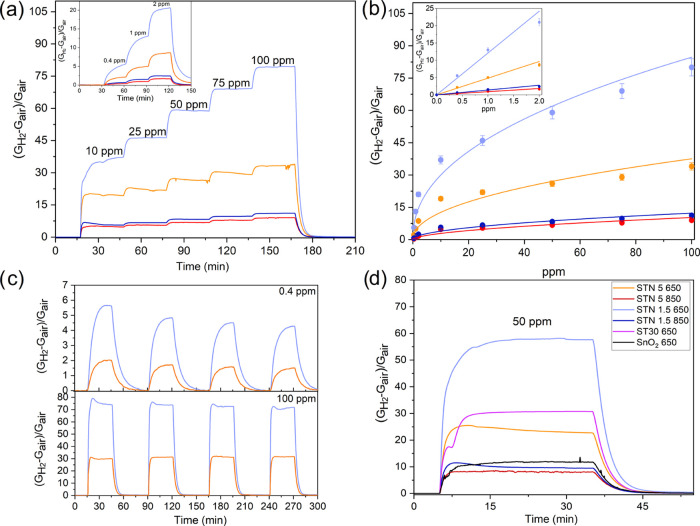
(a) STN film dynamical responses at 450 °C to 0.4, 1, 2 (inset) 10, 25, 50, and 100 ppm of H_2_ in dry air and (b) their calibration curves fitted with a power law function. A linear plot for concentrations lower than 2 ppm (inset) was used to estimate the theoretical LOD. (c) Response to four-cycle injection of 0.4 and 100 ppm of H_2_ as a function of time. (d) Comparison between the responses of STN, ST30 650, and SnO_2_ films to the same concentration of H_2_ (sensors performing at their optimal working temperature of 450 °C for STN and ST30 650 and 400 °C for SnO_2_ 650).^25^ The legend of graph (d) also applies to graphs (a), (b), and (c).

The sensitivity was investigated by exposing the sensors to 0.4, 1, 2, 10, 25, 50, and 100 ppm of H_2_ (Figure 3a). At each H_2_ concentration, the response of STN 650 films was higher than that of STN 850, with STN 1.5 650 overperforming among the others. Figure 3b exhibits the calibration curves for all of the sensors under study. Calibration curves were fitted with a power law function *R*=*ax*^*b*^, *R* being the sensor response and *x* being the gas concentration. The inset in Figure 3b shows that STN 1.5 650 exhibited a significant response even at the lowest concentration of H_2_ injected in the chamber (*R* = 5.55 at 0.4 ppm). Thus, a very low theoretical limit of detection (LOD) is expected. A linear fit (*R* = *cx*) was adopted to better extrapolate the sensor responses at lower concentrations (see the inset in Figure 3b) and to determine the theoretical LOD, which was estimated as in ref (45). The estimated theoretical LOD was 5 ppb, i.e., the lowest LOD obtained so far for MOX gas sensors vs H_2_, to the best of our knowledge. Power law function parameters *a* and *b* and linear fit parameters *c* are listed in Table S2.

The repeatability of the dynamic response vs H_2_ was investigated for the sensors with better sensitivity, namely, STN 1.5 650 and STN 5 650. Figure 3c shows the dynamical responses to 0.4 and 100 ppm of H_2_, i.e., the concentrations extreme of the calibration curves, in four cycles.

The response and recovery times were calculated for 50 ppm (see Figure 3d), and their values are listed in Table S3. The response (about 2 min) and recovery (about 7 min) times were similar for all of the STN except for STN 1.5 650, which showed a slower response and faster recovery kinetics (about 5 min 10 s and 5 min 50 s, respectively). Response and recovery times within minutes have been already observed for traditional SnO_2_ and other materials in our previous works.^11,27,46−48^ They were dependent on the size and geometry of the chamber and on the speed of the gas flow.^27^ Since the filling time of the chamber was about 1 min 15 s, the response times of STN films should be rather fast.

The response of STN films vs H_2_ was compared to SnO_2_ 650 and ST30 650 films in Figure 3d. Ti incorporation in the lattice triplicated the response of pure SnO_2_. The content of Nb in the solid solution and the calcination temperature showed a significant influence on the response. While the 1.5 atom % content of Nb doubled the response of ST30 650, 5 atom % did the opposite. Furthermore, a calcination temperature of 850 °C completely hindered the beneficial effects of both Ti and Nb, resulting in films with a response even lower than SnO_2_ 650.

**Figure 4:**
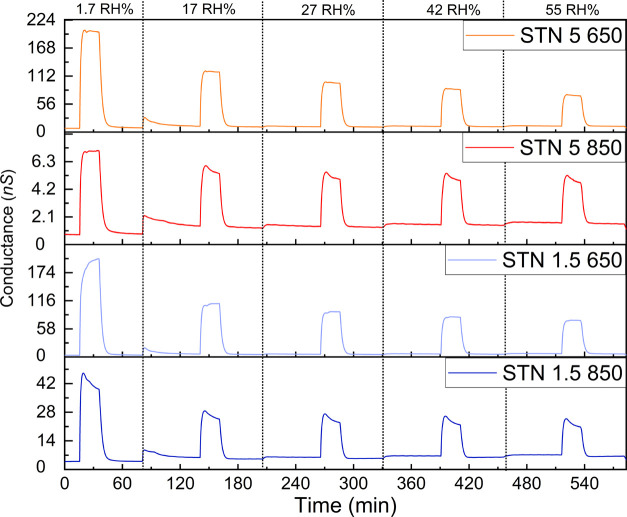
Influence of humidity on the conductance baseline and conductance after injection of 50 ppm of H_2_. The temperature inside the chamber was 29 °C in the whole range of RH %.

The effect of humidity was considered because water vapor can dissociate when interacting with the film, affecting its conductance.^46,49^ To this aim, 50 ppm of H_2_ were injected in the gas chamber at different relative humidity percentages up to 55 RH % (see Figure 4). Water vapor did not significantly change the sensor baselines, but it affected the sensor conductance after injection of H_2_. Figure 4 highlights a significant decrease of the sensor signal up to 17 RH %. At higher humidity levels, the sensor signal to H_2_ kept rather constant, with STN 1.5 650 still sensing at a high level.

**Figure 5:**
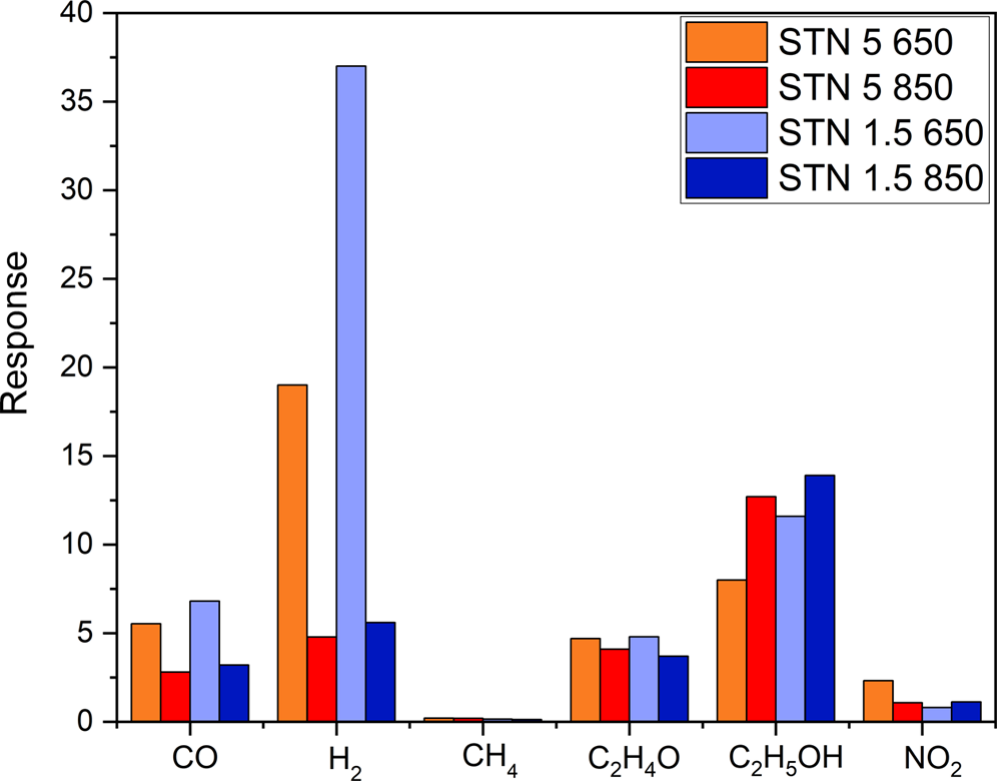
Bar graph of STN film responses (see eqs 1 and 2) to 10 ppm of CO, H_2_, CH_4_, C_2_H_4_O, and C_2_H_5_OH and 1 ppm of NO_2_.

Film selectivity was explored by exposing the sensor to 10 ppm of CO, CH_4_, C_2_H_4_O, and C_2_H_5_OH and 1 ppm of NO_2_. The responses, defined as in eqs 1 and 2 and shown in Figure 5, highlighted that the STN 1.5 650 film was more reactive to hydrogen than to other gases, while the selectivity was lower for STN 5 650, STN 1.5 850, and STN 5 850.

The response of STN 1.5 650 to H_2_ and that of other sensing films based on different metal-oxide semiconductors from the literature are reported in Table 4.

**Table 4:** Comparison of the Hydrogen-Sensing Performance of STN 1.5 650 and STN 5 650 films to Recently Achieved Frequently Used Metal-Oxide Sensors in the Literature^b^^50−55^

material	concentration	response	optimal operating temperature (°C)	LOD	reference
STN 1.5 650	100 ppm	80^a^	450	0.4 ppm/5 ppb^f^	this work
STN 5 650	100 ppm	34^a^	450	0.4 ppm/13 ppb^f^	this work
SnO_2_	100 ppm	15^c^	400		(50)
Pd/SnO_2_	100 ppm	28.5^c^	160	0.25 ppm	(51)
Co/SnO_2_	100 ppm	23^c^	330		(52)
WO_3_	100 ppm	4.8	250	0.25 ppm^f^	(53)
WO_3_–CuO	100 ppm	39	250	0.31 ppm^f^	(53)
CuO	100 ppm	1.7^d^	200	2 ppm	(54)
ZnO	100 ppm	0.95^e^	250		(55)
Ag/ZnO	300 ppm	4.79^e^	250	5 ppm	(55)

a Gas response ,

b Gas response,

c Gas response ,

d Gas response ,

e Gas response , where *G* is the conductance, *R* is the resistance, and *V* is the voltage.

f Indicates theoretical LOD.

STN 1.5 650 performed better than several semiconductors, including highly accredited materials such as SnO_2_ and Pd-decorated and Co-doped SnO_2_,^50−52^ WO_3_,^53^ CuO,^54^ WO_3_–CuO junction,^53^ ZnO, and Ag-doped ZnO.^55^ Moreover, its experimental LOD of 0.4 ppm was far lower than that reported in some recently accredited works.^52,53^ As far as our knowledge is concerned, the best experimental result regarding H_2_ sensing was obtained by Wang et al.,^51^ recording a LOD of 0.25 ppm at *R*_air_/*R*_gas_ = 3. However, the LOD for STN 1.5 650 was 0.4 ppm, but with a response of 5.6.

Based on this experimental result, we concluded that STN 1.5 650 highlights remarkable, still experimentally unexplored, potential for detecting very low concentrations of H_2_. To assess such limits, we used the method described by Huang and Wan.^45^ The theoretical LOD turned out to be as low as 5 ppb, i.e., the lowest theoretical LOD obtained so far by metal-oxide gas sensors.

## Discussion

Since SnO_2_, TiO_2_, and Nb_2_O_5_ are three n-type semiconductors, STN is expected to be n-type as well. The conductance in nanostructured films is the result of bulk and surface properties. The bulk conductance in n-type semiconductors is given by free electrons thermally excited from donor levels near the conduction band. The electron transport in nanostructured films then involves slow percolation through a network of interconnected nanograins surrounded by the atmosphere. When a sensor is exposed to air, at the working temperature between 100 and 500 °C, atmospheric oxygen ionosorbs as molecular (O^2–^) and atomic (O^–^, O^2–^) forms by capturing electrons at a surface state of the semiconductor.^56^ According to the generally accepted conduction model, the presence of negative species on the surface of a metal oxide builds up a depletion layer, whose positive charge exactly compensates for the charge captured at the surface, leading to electroneutrality in the Schottky approximation.^57^ Therefore, in nanostructured semiconductors, the mechanism of conduction is controlled by the number of free carriers and by the potential barrier built as a result of surface charge formation. Arrhenius plots and intergranular energy barrier measurements were conducted to investigate the effect of Nb addition on the conduction mechanism in the Sn- and Ti-oxide solid solution in dry air with and without H_2_. The Arrhenius plot in synthetic air was obtained by changing the temperature from 350 to 900 K at the heating rate of 6 K/min (Figure S8a), while intergranular energy barrier measurements (Figure S8b,c) were conducted as a function of temperature according to the method reported elsewhere.^34^

The conductance of all films increased with temperature because the number of free electrons, thermally excited from ionizable levels under the conduction band, increased. Moreover, STN films were more conductive than ST30 650 and a 5 atom % Nb content resulted in the highest conductance of the films annealed at 650 °C. The higher conductance of STN films can be explained through the substitution of Nb in the lattice, which resulted in extrinsic substitutional doping by introducing additional carriers.^22,24^ This effect may be counteracted by the increase in the energy barrier Δ*E*, which would instead decrease the conductance of the solid solution. As an example, at 450 °C, Δ*E* = 0.9 eV for STN 1.5 650 and STN 5 650 compared to Δ*E* = 0.5 eV for ST30 650. Although it was not possible to quantify the role of Δ*E* on the film conductance, its change was a firm indication of bulk- and surface-defect modifications induced by the addition of Nb. The highest content of Nb and annealing temperature of 850 °C resulted in the lowest conductance for the STN 5 850 film. Indeed, in addition to providing the necessary mobility for Ti and Nb to enter the lattice of SnO_2_, annealing can also induce their segregation as the excess of stress in the lattice is not thermodynamically supported.^58,59^ This process of self-purification would reduce the content of Nb^5+^, negatively impacting the conductance. The energy barriers of STN 1.5 850 and STN 5 850 laid below those of their counterparts annealed at 650 °C, i.e., Δ*E* = 0.7 eV at 450 °C, meaning that annealing changed bulk and surface properties too. Since n-type doping is commonly used to increase the low conductance of TiO_2_ films, the nature of defects that results from Nb addition and their correlation to electron transport in TiO_2_ has been the subject of many studies.^60,61^ Despite the lack of studies on Sn, Ti, and Nb ternary solutions, it is known that the substitution of tetravalent cation (M^4+^) by pentavalent Nb^5+^, as a higher-valence cation, forms either M^3+^ or cation vacancies or both.^60,62^ Trivalent titanium was distinguished in high-resolution scans of Ti 2p core levels. Based on these data, one may infer that the charge compensation of Nb^5+^ in the substitution of Ti^4+^ and Sn^4+^ was achieved by the creation of cation vacancies and trivalent cations and that, as for titania, these additional defects generated new donor states within the band gap, whose energy level depended on the properties of the defect. These defects may be generated in both bulk and surface, with the latter being active sites for redox reactions with the environment.

Under exposure to H_2_, adsorbed oxygen species oxidize the reactive gas generating water and free electrons as in eq 3,^63^ causing conductance increase.
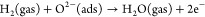
3

As discussed above, the response of STN films and their sensitivity and selectivity were evidently determined by the content of Nb and by the treatment temperature and that deserves a deeper discussion.

First, STN 650 films responded better than their STN 850 counterparts. This can be interpreted in terms of a larger surface area of the former compared to the latter as highlighted by BET measurements (see Table 2). Moreover, the intergrain energy barrier Δ*E* for STN 650 films decreased in the presence of H_2_ while it did not change for the STN 850 ones. It means that the effect of H_2_ on STN 650 conductance was the result of an increase of free charge carriers (see eq 3) and intergrain energy barrier decrease. Indeed, for STN 850, only free charge carriers increase occurred by reaction with H_2_.

Second, it was proved that STN 1.5 650 effectively improved the response of ST30 650 while STN 5 650 did the opposite. Both layers exhibited similar textural properties, thereby, unlike previous considerations, this feature cannot be called forward. However, XPS analyses highlighted that the films differ in surface relative composition of Sn, Ti, and Nb, i.e., 3.8 Nb % for STN 1.5 650 and 9.8 Nb % for STN 5 650. As discussed above, pentavalent Nb at the surface would either affect the reactivity of adsorbed oxygen bound to it or influence the receptive properties of the oxygen adsorbed to neighboring Ti and Sn atoms. Since Nb oxides do not possess qualified properties vs H_2_ detection,^64^ evidently the presence of Nb strengthens the reactivity of neighboring sites. This would explain the improved sensing of STN 1.5 650. Probably, a superficial proportion as high as 10% in Nb at the surface would produce an excess of unreactive oxygen sites that would in turn clear up the beneficial role of Nb on its neighbors.

Third, STN 650 films were more selective to H_2_ than the STN 850 ones, namely, STN 1.5 650 was the most selective to this gas. The main differences between the STN 850 films and STN 650 ones were the specific surface area and the pore size. The specific surface area would only affect the magnitude of the response and not influence the selectivity. Indeed, the pore size may play a significant role as the main free path of the molecules is comparable to the length of the pores.^46,65^ The pore size may affect the selectivity as is small enough to make the diffusion properties of the gases in the film dependent on their molecular diameter and mass. The pore size of the STN 650 samples is smaller than that of the STN 850 ones, e.g., 16.7 nm for STN 1.5 650 and 24.0 nm for STN 1.5 850. In this way, the STN 650 films would become more selective to H_2_ because H_2_ molecules diffuse deeper into the films than much larger molecules such as C_2_H_4_O and C_2_H_5_OH, thereby enabling a relatively wide surface for its sensing.

## Conclusions

This work investigated the effect of the addition of Nb to a solid solution of Sn and Ti oxides with regards to morphological, structural, and electrical properties and particularly to H_2_ sensing. Films with Nb 1.5 and 5 atom % contents were synthesized through the sol–gel technique by keeping the Sn–Ti ratio constant at the optimal value for sensing performance of the solid solution without Nb, i.e., Sn/Ti = 70:30.^25^ The influence of calcination temperature was sought by heating the powders at 650 and 850 °C. SEM observations performed on the powders revealed morphologies consisting of spheroidal nanograins. Particle-size distributions of STN 5 650 and STN 5 850 were analogous, while STN 1.5 650 showed a more compact distribution and in a range of smaller sizes than STN 1.5 850. A high content of Nb prevented grain coalescence at high temperatures but made the growth of particles inhomogeneous during the synthesis. The annealing treatment strongly influenced the BET surface area of the powders, which was larger for the samples treated at 650 °C (STN 1.5 650 = 43.7 m^2^/g) compared to that of STN 1.5 850 (22.6 m^2^/g). XRPD analysis showed that Nb and Ti partly entered the bulk of rutile-type SnO_2_, forming a solid solution of (Sn,Ti,Nb)*_x_*O_2_. A small percentage of anatase-type structure (3 wt %), mainly ascribed to titania, was also detected. The remaining Nb and Ti segregated at the surface of the nanograins, as indicated by XPS. Segregation was more conspicuous for the highest content of niobium after annealing at 850 °C. According to high-resolution scans of Nb 3d core levels, Nb was located as substitutional in Ti and Sn sites, assuming a pentavalent nature. Moreover, the substitution of tetravalent cation by pentavalent Nb^5+^ may generate additional defects, which impact the surface reactivity.

The electric properties were significantly modified by the addition of Nb, inasmuch as it increased the conductance of all of the solid solutions by a factor of 3 to 10.

The sensing performance was probed by investigating sensitivity, repeatability, and selectivity. The content of Nb in the solid solution and the calcination temperature showed a significant influence on the response. While a 1.5 atom % content of Nb doubled the response to H_2_ of ST30 650, 5 atom % did the opposite. Furthermore, the calcination temperature at 850 °C completely hindered the beneficial effect of both Ti and Nb, resulting in films with a response even lower than SnO_2_ 650. Despite STN 1.5 650 showed the highest response toward H_2_, the responses toward the other gases were comparable to that of the other films. This made it the most selective of the films tested. Moreover, at high humidity levels, only STN 1.5 650 was still sensing at a very high level.

In conclusion, the films made with the powders annealed at 650 °C with a 1.5 atom % content of Nb turned out to be the most interesting materials for H_2_ sensing. By comparing the performance to other metal-oxide films in the literature, it emerged that STN 1.5 650 exhibited an extremely high response, even at the lowest concentration of H_2_ (*R* = 5.6 toward 0.4 ppm). A very low theoretical limit of detection was estimated according to the method in ref (45). It resulted in extraordinary capability to sense as low a concentration as a LOD of 5 ppb.

## Supporting Information Available

In-depth description of material characterization methods and gas sensor preparation; morphological, textural, and structural characterizations; response variation according to the temperature change; fit parameters for calibration curves; response and recovery times; and film conductance vs temperature and energy barrier measurement (PDF)

## References

[ref1] Basu A. K.; Tatiya S.; Bhatt G.; Bhattacharya S. Fabrication Processes for Sensors for Automotive Applications: A Review. In Sensors for Automotive and Aerospace Applications; Springer, 2019; 123–142.

[ref2] Chauhan P. S.; Bhattacharya S. Hydrogen Gas Sensing Methods, Materials, and Approach to Achieve Parts per Billion Level Detection: A Review. Int. J. Hydrogen Energy 2019, 44, 26076–26099. 10.1016/j.ijhydene.2019.08.052.

[ref3] Zappi A.; Hernandez R.; Holmes W. E. A Review of Hydrogen Production from Anaerobic Digestion. Int. J. Environ. Sci. Technol. 2021, 18, 4075–4090. 10.1007/s13762-020-03117-w.

[ref4] Richter D.; Fried A.; Wert B. P.; Walega J. G.; Tittel F. K. Development of a Tunable Mid-IR Difference Frequency Laser Source for Highly Sensitive Airborne Trace Gas Detection. Appl. Phys. B: Lasers Opt. 2002, 75, 281–288. 10.1007/s00340-002-0948-y. 12599397

[ref5] Uslu H.; Büyükpınar Ç.; Unutkan T.; Serbest H.; SAN N.; Turak F.; Bakırdere S. A Novel Analytical Method for Sensitive Determination of Lead: Hydrogen Assisted T-Shape Slotted Quartz Tube-Atom Trap-Flame Atomic Absorption Spectrometry. Microchem. J. 2018, 137, 155–159. 10.1016/j.microc.2017.10.015.

[ref6] Kamiński M.; Kartanowicz R.; Jastrzębski D.; Kamiński M. M. Determination of Carbon Monoxide, Methane and Carbon Dioxide in Refinery Hydrogen Gases and Air by Gas Chromatography. J. Chromatogr. A 2003, 989, 277–283. 10.1016/S0021-9673(03)00032-3. 12650260

[ref7] Farrah D.; Bernard-Salas J.; Spoon H. W. W.; Soifer B. T.; Armus L.; Brandl B.; Charmandaris V.; Desai V.; Higdon S.; Devost D.; Houck J. High-Resolution Mid-Infrared Spectroscopy of Ultraluminous Infrared Galaxies. Astrophys. J. 2007, 667, 149–169. 10.1086/520834.

[ref8] Sears J.; Rogers T.; McCoskey J.; Lockrem L.; Watts H.; Pingel L.; Conca J. Proton Transfer Reaction Mass Spectrometry as a Real-Time Method for Continuous Soil Organic Vapor Detection. In Continuous Soil Gas Measurements: Worst Case Risk Parameters; ASTM International: 100 Barr Harbor Drive, PO Box C700, West Conshohocken, PA 19428-2959, 2013; pp 32–44. https://doi.org/10.1520/STP157020130026.

[ref9] G N. First Fifty Years of Chemoresistive Gas Sensors. Chemosensors 2015, 3, 1–20. 10.3390/chemosensors3010001.

[ref10] Valt M.; Fabbri B.; Gaiardo A.; Gherardi S.; Casotti D.; Cruciani G.; Pepponi G.; Vanzetti L.; Iacob E.; Malagù C.; Bellutti P.; Guidi V. Aza-Crown-Ether Functionalized Graphene Oxide for Gas Sensing and Cation Trapping Applications. Mater. Res. Express 2019, 6, 075603 10.1088/2053-1591/ab11fb.

[ref11] Gaiardo A.; Fabbri B.; Guidi V.; Bellutti P.; Giberti A.; Gherardi S.; Vanzetti L.; Malagù C.; Zonta G. Metal Sulfides as Sensing Materials for Chemoresistive Gas Sensors. Sensors 2016, 16, 296 10.3390/s16030296. 26927120 PMC4813871

[ref12] Tricoli A.; Righettoni M.; Pratsinis S. E. Minimal Cross-Sensitivity to Humidity during Ethanol Detection by SnO_2_–TiO_2_ Solid Solutions. Nanotechnology 2009, 20, 315502 10.1088/0957-4484/20/31/315502. 19597246

[ref13] Pargoletti E.; Verga S.; Chiarello G. L.; Longhi M.; Cerrato G.; Giordana A.; Cappelletti G. Exploring Sn_x_Ti_1–x_O_2_ Solid Solutions Grown onto Graphene Oxide (GO) as Selective Toluene Gas Sensors. Nanomaterials 2020, 10, 761 10.3390/nano10040761. PMC7221561 32326649

[ref14] Radecka M.; Zakrzewska K.; Rękas M. SnO_2_–TiO_2_ Solid Solutions for Gas Sensors. Sens. Actuators, B 1998, 47, 194–204. 10.1016/S0925-4005(98)00023-9.

[ref15] Carotta M. C.; Gherardi S.; Guidi V.; Malagù C.; Martinelli G.; Vendemiati B.; Sacerdoti M.; Ghiotti G.; Morandi S. Electrical and Spectroscopic Properties of Ti_0.2_Sn_0.8_O_2_ Solid Solution for Gas Sensing. Thin Solid Films 2009, 517, 6176–6183. 10.1016/j.tsf.2009.04.002.

[ref16] Carotta M. C.; Fioravanti A.; Gherardi S.; Malagù C.; Sacerdoti M.; Ghiotti G.; Morandi S. (Ti,Sn) Solid Solutions as Functional Materials for Gas Sensing. Sens. Actuators, B 2014, 194, 195–205. 10.1016/j.snb.2013.12.021.

[ref17] Carney C. M.; Yoo S.; Akbar S. A. TiO_2_–SnO_2_ Nanostructures and Their H_2_ Sensing Behavior. Sens. Actuators, B 2005, 108, 29–33. 10.1016/j.snb.2004.11.058.

[ref18] Shi Y.; Xu H.; Liu T.; Zeb S.; Nie Y.; Zhao Y.; Qin C.; Jiang X. Advanced Development of Metal Oxide Nanomaterials for H_2_ Gas Sensing Applications. Mater. Adv. 2021, 2, 1530–1569. 10.1039/D0MA00880J.

[ref19] Zeng W.; Liu T.; Wang Z.; Tsukimoto S.; Saito M.; Ikuhara Y. Selective Detection of Formaldehyde Gas Using a Cd-Doped TiO_2_-SnO_2_ Sensor. Sensors 2009, 9, 9029–9038. 10.3390/s91109029. 22291551 PMC3260628

[ref20] Carotta M. C.; Cervi A.; Giberti A.; Guidi V.; Malagù C.; Martinelli G.; Puzzovio D. Metal-Oxide Solid Solutions for Light Alkane Sensing. Sens. Actuators, B 2008, 133, 516–520. 10.1016/j.snb.2008.03.012.

[ref21] Carotta M. C.; Guidi V.; Malagù C.; Vendemiati B.; Zanni A.; Martinelli G.; Sacerdoti M.; Licoccia S.; Vona M. L. Di.; Traversa E. Vanadium and Tantalum-Doped Titanium Oxide (TiTaV): A Novel Material for Gas Sensing. Sens. Actuators, B 2005, 108, 89–96. 10.1016/j.snb.2004.11.070.

[ref22] Ferroni M.; Carotta M.; Guidi V.; Martinelli G.; Ronconi F.; Richard O.; Van Dyck D.; Van Landuyt J. Structural Characterization of Nb–TiO_2_ Nanosized Thick-Films for Gas Sensing Application. Sens. Actuators, B 2000, 68, 140–145. 10.1016/S0925-4005(00)00474-3.

[ref23] Guidi V.; Carotta M. C.; Ferroni M.; Martinelli G.; Sacerdoti M. Effect of Dopants on Grain Coalescence and Oxygen Mobility in Nanostructured Titania Anatase and Rutile. J. Phys. Chem. B 2003, 107, 120–124. 10.1021/jp013572u.

[ref24] Gardecka A. J.; Goh G. K. L.; Sankar G.; Parkin I. P. On the Nature of Niobium Substitution in Niobium Doped Titania Thin Films by AACVD and Its Impact on Electrical and Optical Properties. J. Mater. Chem. A 2015, 3, 17755–17762. 10.1039/c5ta03772g.

[ref25] Carotta M. C.; Benetti M.; Guidi V.; Gherardi S.; Malagu’ C.; Vendemiati B.; Martinelli G. Nanostructured (Sn,Ti,Nb)O_2_ Solid Solution for Hydrogen Sensing. MRS Proc. 2006, 915, 0915–R07-10. 10.1557/PROC-0915-R07-10.

[ref26] Chiorino A.; Ghiotti G.; Prinetto F.; Carotta M.; Gnani D.; Martinelli G. Preparation and Characterization of SnO_2_ and MoO_x_–SnO_2_ Nanosized Powders for Thick Film Gas Sensors. Sens. Actuators, B 1999, 58, 338–349. 10.1016/S0925-4005(99)00094-5.

[ref27] Gaiardo A.; Fabbri B.; Giberti A.; Valt M.; Gherardi S.; Guidi V.; Malagù C.; Bellutti P.; Pepponi G.; Casotti D.; Cruciani G.; Zonta G.; Landini N.; Barozzi M.; Morandi S.; Vanzetti L.; Canteri R.; Della Ciana M.; Migliori A.; Demenev E. Tunable Formation of Nanostructured SiC/SiOC Core-Shell for Selective Detection of SO_2_. Sens. Actuators, B 2020, 305, 127485 10.1016/j.snb.2019.127485.

[ref28] Guidi V.; Fabbri B.; Gaiardo A.; Gherardi S.; Giberti A.; Malagù C.; Zonta G.; Bellutti P. Metal Sulfides as a New Class of Sensing Materials. Procedia Eng. 2015, 120, 138–141. 10.1016/j.proeng.2015.08.586.

[ref29] Gaiardo A.; Fabbri B.; Giberti A.; Guidi V.; Bellutti P.; Malagù C.; Valt M.; Pepponi G.; Gherardi S.; Zonta G.; Martucci A.; Sturaro M.; Landini N. ZnO and Au/ZnO Thin Films: Room-Temperature Chemoresistive Properties for Gas Sensing Applications. Sens. Actuators, B 2016, 237, 1085–1094. 10.1016/j.snb.2016.07.134.

[ref30] Zonta G.; Astolfi M.; Casotti D.; Cruciani G.; Fabbri B.; Gaiardo A.; Gherardi S.; Guidi V.; Landini N.; Valt M.; Malagù C. Reproducibility Tests with Zinc Oxide Thick-Film Sensors. Ceram. Int. 2020, 46, 6847–6855. 10.1016/j.ceramint.2019.11.178.

[ref31] Agents C. Commission Directive (EU) 2017/164. Off. J. Eur. Union 2017, 1989, 115–120.

[ref32] Zhang H.; Sun Z.; Hu Y. H. Steam Reforming of Methane: Current States of Catalyst Design and Process Upgrading. Renewable Sustainable Energy Rev. 2021, 149, 111330 10.1016/j.rser.2021.111330.

[ref33] Wilhelm D.; Simbeck D.; Karp A.; Dickenson R. Syngas Production for Gas-to-Liquids Applications: Technologies, Issues and Outlook. Fuel Process. Technol. 2001, 71, 139–148. 10.1016/S0378-3820(01)00140-0.

[ref34] Lantto V.; Rompplainen P.; Leppävuori S. A Study of the Temperature Dependence of the Barrier Energy in Porous Tin Dioxide. Sens. Actuators 1988, 14, 149–163. 10.1016/0250-6874(88)80062-3.

[ref35] International Organization for Standardization. Determination of the Specific Surface Area of Solids by Gas Adsorption—BET Method (ISO 9277:2010(E)); ISO, 2010; Vol. 9277, p 30.

[ref36] Hirata T. Oxygen Position, Octahedral Distortion, and Bond-Valence Parameter from Bond Lengths in Ti_1–x_Sn_x_O_2_ (0≤ x≤1). J. Am. Ceram. Soc. 2000, 83, 3205–3207. 10.1111/j.1151-2916.2000.tb01706.x.

[ref37] Howard C. J.; Sabine T. M.; Dickson F. Structural and Thermal Parameters for Rutile and Anatase. Acta Crystallogr., Sect. B: Struct. Sci. 1991, 47, 462–468. 10.1107/S010876819100335X.

[ref38] Shannon R. D. Revised Effective Ionic Radii and Systematic Studies of Interatomic Distances in Halides and Chalcogenides. Acta Crystallogr., Sect. A: Cryst. Phys., Diffr., Theor. Gen. Crystallogr. 1976, 32, 751–767. 10.1107/S0567739476001551.

[ref39] Jung W.; Tuller H. L. Investigation of Surface Sr Segregation in Model Thin Film Solid Oxidefuel Cell Perovskite Electrodes. Energy Environ. Sci. 2012, 5, 5370–5378. 10.1039/C1EE02762J.

[ref40] Hamada I.; Uozumi A.; Morikawa Y.; Yanase A.; Katayama-Yoshida H. A Density Functional Theory Study of Self-Regenerating Catalysts LaFe_1– x_M_x_O_3–y_ (M = Pd, Rh, Pt). J. Am. Chem. Soc. 2011, 133, 18506–18509. 10.1021/ja110302t. 22026920

[ref41] Farva U.; Kim J. Growth Temperature-Dependent Morphological, Optical, and Electrical Study of SnO_2_ Thin Film by Atomic Layer Deposition. Mater. Chem. Phys. 2021, 267, 124584 10.1016/j.matchemphys.2021.124584.

[ref42] Chen Q. Nb_2_O_5_ Improved Photoluminescence, Magnetic and Faraday Rotation Properties of Magneto-Optical Glasses. J. Non-Cryst. Solids 2019, 519, 119451 10.1016/j.jnoncrysol.2019.05.027.

[ref43] Xu Y.; Wu S.; Wan P.; Sun J.; Hood Z. D. Introducing Ti^3+^ Defects Based on Lattice Distortion for Enhanced Visible Light Photoreactivity in TiO_2_ Microspheres. RSC Adv. 2017, 7, 32461–32467. 10.1039/C7RA04885H.

[ref44] Di Valentin C.; Pacchioni G.; Selloni A. Reduced and N-Type Doped TiO_2_: Nature of Ti^3+^ Species. J. Phys. Chem. C 2009, 113, 20543–20552. 10.1021/jp9061797.

[ref45] Huang J.; Wan Q. Gas Sensors Based on Semiconducting Metal Oxide One-Dimensional Nanostructures. Sensors 2009, 9, 9903–9924. 10.3390/s91209903. 22303154 PMC3267202

[ref46] Spagnoli E.; Krik S.; Fabbri B.; Valt M.; Ardit M.; Gaiardo A.; Vanzetti L.; Della Ciana M.; Cristino V.; Vola G.; Caramori S.; Malagù C.; Guidi V. Development and Characterization of WO_3_ Nanoflakes for Selective Ethanol Sensing. Sens. Actuators, B 2021, 347, 130593 10.1016/j.snb.2021.130593.

[ref47] Gaiardo A.; Zonta G.; Gherardi S.; Malagù C.; Fabbri B.; Valt M.; Vanzetti L.; Landini N.; Casotti D.; Cruciani G.; Della Ciana M.; Guidi V. Nanostructured SmFeO_3_ Gas Sensors: Investigation of the Gas Sensing Performance Reproducibility for Colorectal Cancer Screening. Sensors 2020, 20, 5910 10.3390/s20205910. PMC7589820 33086770

[ref48] Valt M.; Caporali M.; Fabbri B.; Gaiardo A.; Krik S.; Iacob E.; Vanzetti L.; Malagù C.; Banchelli M.; D’Andrea C.; Serrano-Ruiz M.; Vanni M.; Peruzzini M.; Guidi V. Air Stable Nickel-Decorated Black Phosphorus and Its Room-Temperature Chemiresistive Gas Sensor Capabilities. ACS Appl. Mater. Interfaces 2021, 13, 44711–44722. 10.1021/acsami.1c10763. 34506713 PMC8461602

[ref49] Madou M. J.; Morrison S. R. Gas Sensors Based on Semiconductor Powders. In Chemical Sensing with Solid State Devices; Elsevier, 1989; Vol. 8, pp 479–516. https://doi.org/10.1016/B978-0-12-464965-1.50017-X.

[ref50] Umar A.; Ammar H. Y.; Kumar R.; Almas T.; Ibrahim A. A.; AlAssiri M. S.; Abaker M.; Baskoutas S. Efficient H_2_ Gas Sensor Based on 2D SnO_2_ Disks: Experimental and Theoretical Studies. Int. J. Hydrogen Energy 2020, 45, 26388–26401. 10.1016/j.ijhydene.2019.04.269.

[ref51] Wang F.; Hu K.; Liu H.; Zhao Q.; Wang K.; Zhang Y. Low Temperature and Fast Response Hydrogen Gas Sensor with Pd Coated SnO_2_ Nanofiber Rods. Int. J. Hydrogen Energy 2020, 45, 7234–7242. 10.1016/j.ijhydene.2019.12.152.

[ref52] Liu L.; Guo C.; Li S.; Wang L.; Dong Q.; Li W. Improved H_2_ Sensing Properties of Co-Doped SnO_2_ Nanofibers. Sens. Actuators, B 2010, 150, 806–810. 10.1016/j.snb.2010.07.022.

[ref53] Ding W.; Ansari N.; Yang Y.; Bachagha K. Superiorly Sensitive and Selective H_2_ Sensor Based on P-n Heterojunction of WO_3_–CoO Nanohybrids and Its Sensing Mechanism. Int. J. Hydrogen Energy 2021, 46, 28823–28837. 10.1016/j.ijhydene.2021.06.070.

[ref54] Nakate U. T.; Lee G. H.; Ahmad R.; Patil P.; Hahn Y.-B.; Yu Y. T.; Suh E. Nano-Bitter Gourd like Structured CuO for Enhanced Hydrogen Gas Sensor Application. Int. J. Hydrogen Energy 2018, 43, 22705–22714. 10.1016/j.ijhydene.2018.09.162.

[ref55] Agarwal S.; Kumar S.; Agrawal H.; Moinuddin M. G.; Kumar M.; Sharma S. K.; Awasthi K. An Efficient Hydrogen Gas Sensor Based on Hierarchical Ag/ZnO Hollow Microstructures. Sens. Actuators, B 2021, 346, 130510 10.1016/j.snb.2021.130510.

[ref56] Barsan N.; Rebholz J.; Weimar U. Conduction Mechanism Switch for SnO_2_ Based Sensors during Operation in Application Relevant Conditions; Implications for Modeling of Sensing. Sens. Actuators, B 2015, 207, 455–459. 10.1016/j.snb.2014.10.016.

[ref57] Barsan N.; Weimar U. Conduction Model of Metal Oxide Gas Sensors. J. Electroceram. 2001, 7, 143–167. 10.1023/a:1014405811371.

[ref58] Arbiol J.; Cerdà J.; Dezanneau G.; Cirera A.; Peiró F.; Cornet A.; Morante J. R. Effects of Nb Doping on the TiO_2_ Anatase-to-Rutile Phase Transition. J. Appl. Phys. 2002, 92, 853–861. 10.1063/1.1487915.

[ref59] Uyanga E.; Gibaud A.; Daniel P.; Sangaa D.; Sevjidsuren G.; Altantsog P.; Beuvier T.; Lee C. H.; Balagurov A. M. Structural and Vibrational Investigations of Nb-Doped TiO_2_ Thin Films. Mater. Res. Bull. 2014, 60, 222–231. 10.1016/j.materresbull.2014.08.035.

[ref60] Ruiz A. M.; Dezanneau G.; Arbiol J.; Cornet A.; Morante J. R. Insights into the Structural and Chemical Modifications of Nb Additive on TiO_2_ Nanoparticles. Chem. Mater. 2004, 16, 862–871. 10.1021/cm0351238.

[ref61] Zeng W.; Liu T.; Wang Z. Impact of Nb Doping on Gas-Sensing Performance of TiO_2_ Thick-Film Sensors. Sens. Actuators, B 2012, 166–167, 141–149. 10.1016/j.snb.2012.02.016.

[ref62] Pan X.; Yang M.-Q.; Fu X.; Zhang N.; Xu Y.-J. Defective TiO_2_ with Oxygen Vacancies: Synthesis, Properties and Photocatalytic Applications. Nanoscale 2013, 5, 3601–3614. 10.1039/c3nr00476g. 23532413

[ref63] Koo W.-T.; Cho H.-J.; Kim D.-H.; Kim Y. H.; Shin H.; Penner R. M.; Kim I.-D. Chemiresistive Hydrogen Sensors: Fundamentals, Recent Advances, and Challenges. ACS Nano 2020, 14, 14284–14322. 10.1021/acsnano.0c05307. 33124428

[ref64] Mokrushin A. S.; Simonenko T. L.; Simonenko N. P.; Gorobtsov P. Y.; Kadyrov N. C.; Simonenko E. P.; Sevastyanov V. G.; Kuznetsov N. T. Chemoresistive Gas-Sensing Properties of Highly Dispersed Nb_2_O_5_ Obtained by Programmable Precipitation. J. Alloys Compd. 2021, 868, 159090 10.1016/j.jallcom.2021.159090.

[ref65] Guo Y.; He X.; Huang W.; Wang M. Microstructure Effects on Effective Gas Diffusion Coefficient of Nanoporous Materials. Transp. Porous Media 2019, 126, 431–453. 10.1007/s11242-018-1165-4.

