# Supplementary material for: Design of a Metal-Oxide Solid Solution for Sub-ppm H2 Detection
Source: ACS Sens. 2022 Feb 16;7(2):573–83. doi: 10.1021/acssensors.1c02481 (PMC8886563; doi:10.1021/acssensors.1c02481)
Supplement: Supplementary file 1 — se1c02481_si_001.pdf [file se1c02481_si_001.pdf]

Supporting Information

Design of a metal-oxide solid solution for sub-ppm H_2 detection

Elena Spagnoli[1,†,*], Andrea Gaiardo[2,†,*], Barbara Fabbri[1], Matteo Valt[2], Soufiane Krik[1,3], Matteo Ardit[1], Giuseppe Cruciani[1], Michele Della Ciana[1,4], Lia Vanzetti[2], Gabriele Vola[5], Sandro Gherardi[1], Pierluigi Bellutti[2], Cesare Malagù[1] , Vincenzo Guidi[1]

1) Department of Physics and Earth Sciences – University of Ferrara, via Giuseppe Saragat 1, Ferrara 44122, Italy

2) MNF- Micro Nano Facility Sensors and Devices Center, Bruno Kessler Foundation, via Sommarive 18, Trento 38123, Italy

3) Sensing Technologies Lab, Faculty of Science and Technology, Free University of Bozen-Bolzano, piazza Università 1, Bolzano 39100, Italy

4) National Research Council, Institute for Microelectronics and Microsystems, via Gobetti 101, Bologna 40129, Italy

5) Cimprogetti S.r.l. Lime Technologies, via Pasubio, Bergamo 24044, Italy

* E-mails: elena.spagnoli@unife.it; gaiardo@fbk.eu

† These authors contributed equally to this work

Keywords: $(Sn,Ti,Nb)_xO_2$; metal oxides solid solution; chemoresistive gas sensors; H_2 detection; nanostructured MOX.

Material characterization

The chemical composition and the morphology of the synthesized powders were investigated by Energy Dispersion X-Ray spectroscopy (EDX) and Scanning Electron Microscopy (SEM), using a Zeiss EVO 40 microscope. Gas porosity and specific surface area of the powders were investigated using a Micromeritics TriStar II Plus automated gas sorptometer, with nominal resolution of > 0.01 m^2/g. The running program of N_2 adsorption-desorption isotherm consisted of 83 points of relative pressure, from 0.05 (p/p_0) up to 1.00 (p/p_0) and back, being p the pressure of the adsorptive in equilibrium with the adsorbate and p_0 the saturation vapour pressure of the adsorptive. The specific surface area was determined according to Brunauer-Emmett-Teller Specific Surface Area (BET-SSA) theory.[2] Pore volume and pore-size distribution (PSD) were determined according to Adsorption/Desorption Barrett-Joyner-Halenda (BJH) method and to the method of non-local density functional theory (DFT), respectively. The particle size analysis was performed by exploiting ImageJ 2 software. X-ray powder diffraction (XRD) data were collected at room temperature on a Bruker D8 Advance Da Vinci diffractometer working in Bragg-Brentano geometry, and equipped with a LynxEye XE silicon strip detector (angular range of the detector window size = 2.585° 2θ) set to discriminate Cu Kα1,2 radiation. Qualitative phase analysis of collected patterns was performed by means of the Bruker AXS EVA software (v.5) and refined by means of the fundamental-parameter approach (TOPAS v.5.0). X-ray Photoelectron Spectroscopy (XPS) analyses were performed using Kratos AXIS UltraDLD instrument (Kratos Analytical, Manchester, UK) equipped with a hemispherical analyser and a monochromatic Al Kα (1486.6 eV) X-ray source, in spectroscopy mode. The analyses were performed with a take-off angle between the analyzer axis and the normal to the sample surface of 0°. For the measurements, the powders were attached to the sample holder using double-sided carbon tape. For each sample, the survey, the high-resolution scans of the Sn 3d, Ti 2p and Nb 3d core levels were collected. XPS quantification was performed using the instrument sensitivity factors and the high-resolution scans. Charge compensation was achieved using a flood gun and all core levels were referenced to the C-C/C-H component in C 1s at 285.0 eV. All XPS data were analysed using the software described in Speranza and Canteri.[1]

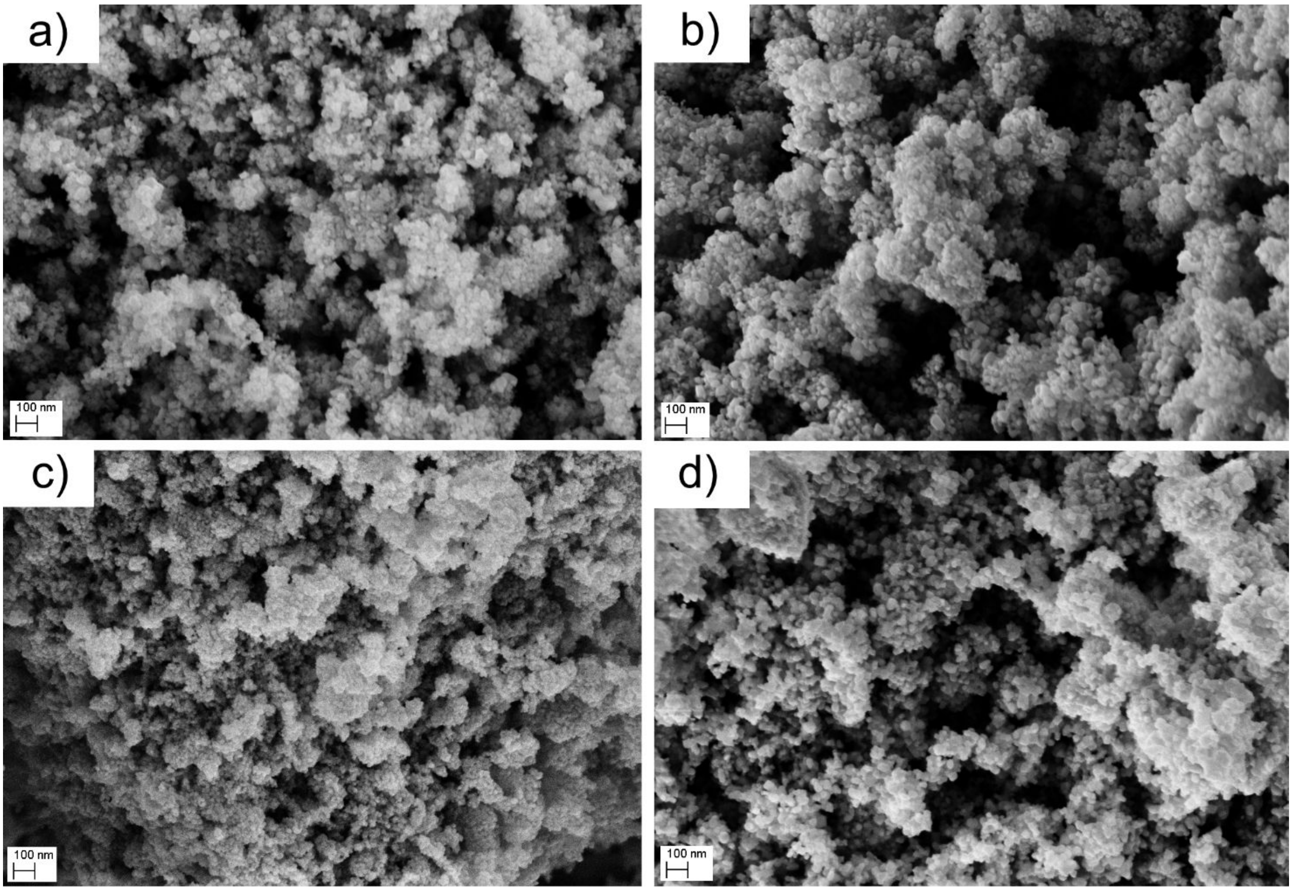

Figure S1. SEM images of STN 5 powders fired at **a)** 650°C and **b)** 850°C and STN 1.5 powders fired at **c)** 650°C and **d)** 850°C, highlighting spheroidal morphology of the nanostructures. In **c)**, the STN 1.5 650 sample shows the smallest particle size distribution.

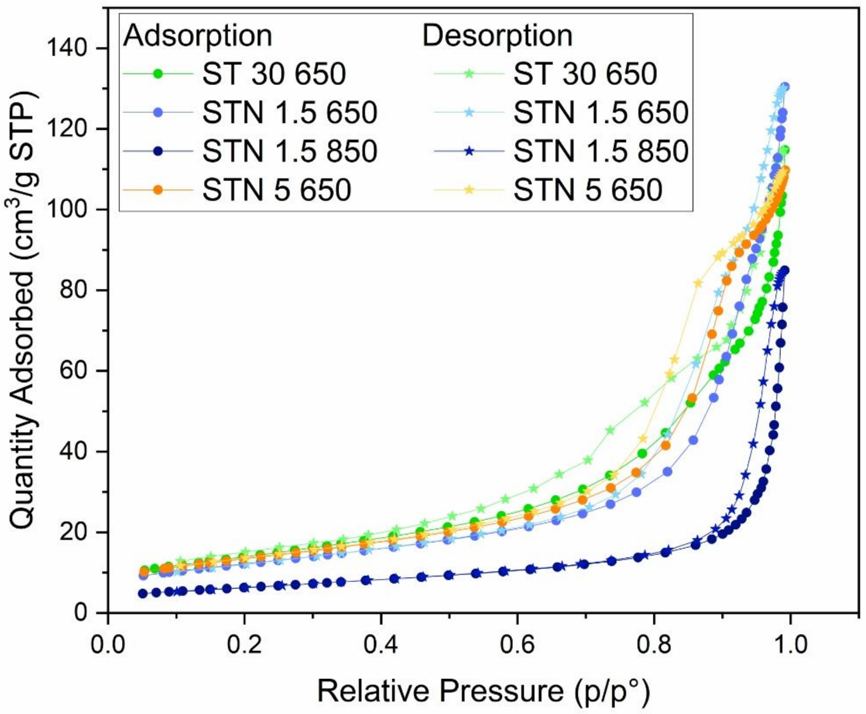

Figure S2. N_2 isotherm profile of samples ST 30 650, STN 1.5 650, STN 1.5 850 and STN 5 650

Table S1. Quantitative phase analysis and Rutile-type unit-cell parameters and crystal-size with their standard deviations for the STN samples collected at *RT*. The R-weighted pattern refinement agreement factors were $R_{WP\ (ST\ 650)}$ =0.094, $R_{WP\ (STN\ 1.5\ 650)}$ =0.110, $R_{WP\ (STN\ 5\ 650)}$ =0.117, $R_{WP\ (STN\ 1.5\ 850)}$ =0.112 and $R_{WP\ (STN\ 5\ 850)}$ =0.118, indicating the good quality of the fit.

	Quantitative phase analysis	*Rutile-type phase: unit-cell parameters & Crystal size*			
Sample	rutile-/anatase-type wt%	a(nm)	c(nm)	V(nm^3)	Crystallite size (nm)
ST30 650	99.1(2)/0.9(2)	0.47040(3)	0.31488(3)	0.06968(1)	7.4(1)
STN 1.5 650	97.3(3)/2.7(3)	0.47070(3)	0.31460(3)	0.06970(1)	9.4(2)
STN 5 650	97.1(3)/2.9(3)	0.47190(3)	0.31619(3)	0.07041(1)	8.8(2)
STN 1.5 850	97.3(3)/2.7(3)	0.47077(3)	0.31463(4)	0.06973(1)	7.9(1)
STN 5 850	99.0(2)/1.0(2)	0.47148(2)	0.31576(3)	0.07019(1)	12.8(3)

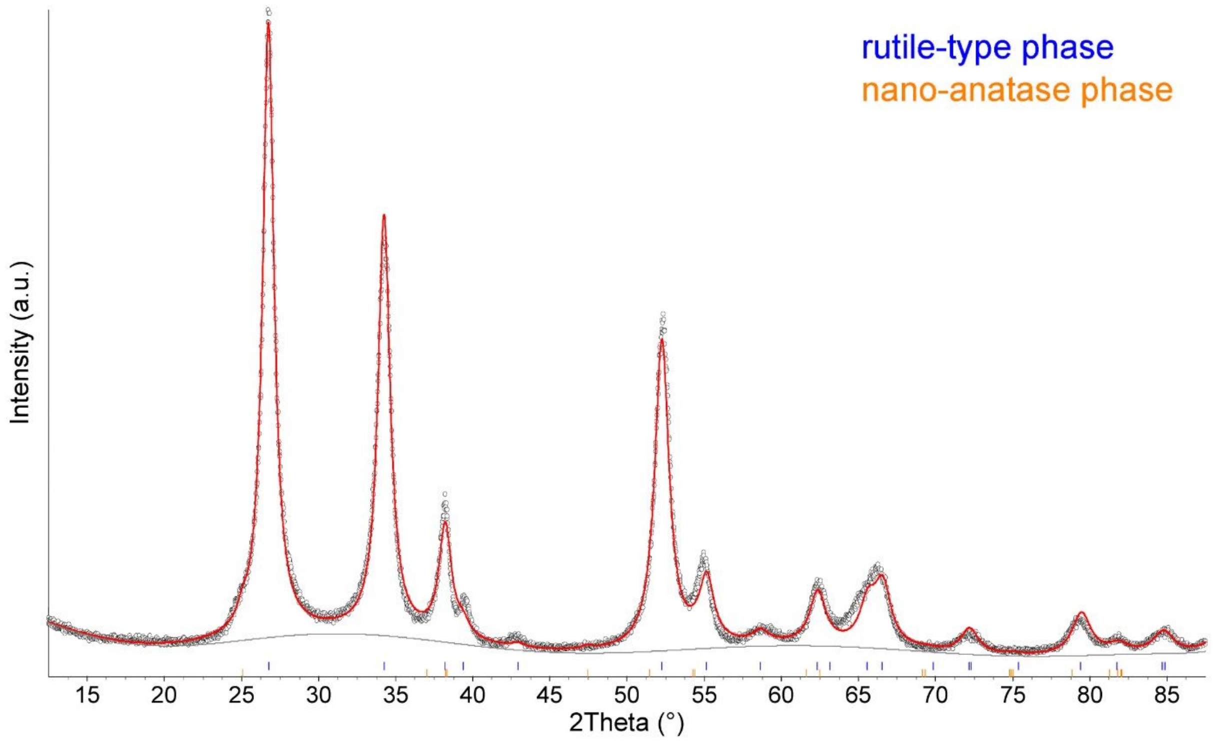

Figure S3. Whole refinement for STN 1.5 650 plot. The experimental profile is represented by black dots, the best-fit refinement profile is the continuous red line, and the background fit is the grey curve Vertical ticks mark the position of reflections for rutile type (blue) and for anatase-type (orange) phases.

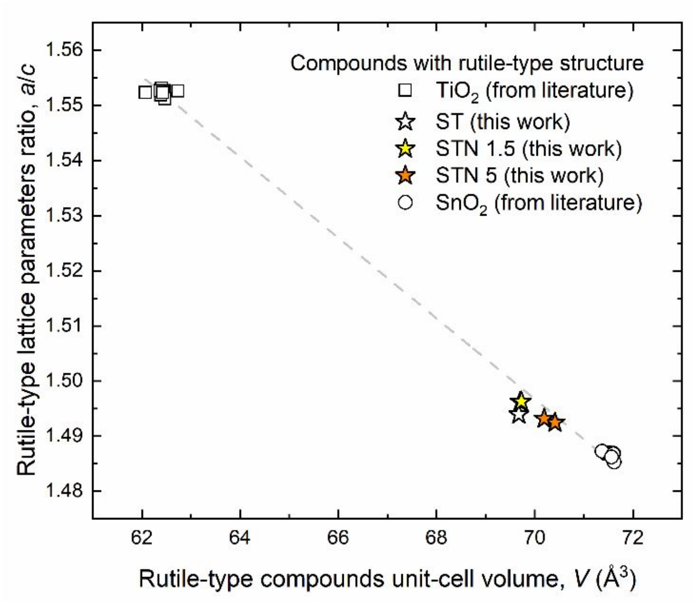

Figure S4. Lattice parameter ratio (a/c) vs. the unit-cell volume V for rutile-type crystalline structure. Open squares and circles refer to data of TiO_2 rutile and SnO_2 cassiterite phases respectively[3–14] while white, yellow and orange stars refer to data of the STN and the ST samples in this work.

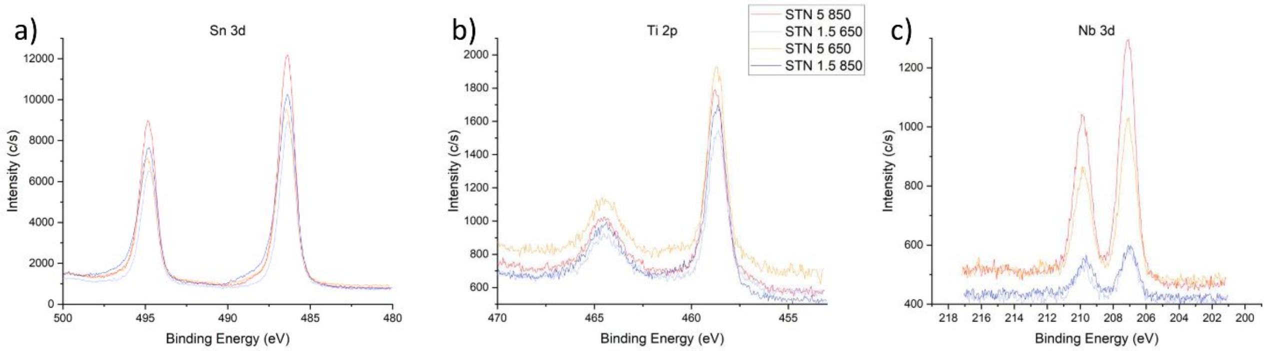

Figure S5. High-resolution scans of **a)** Sn 3d, **b)** Ti 2p and **c)** Nb 3d core levels for all the STN samples.

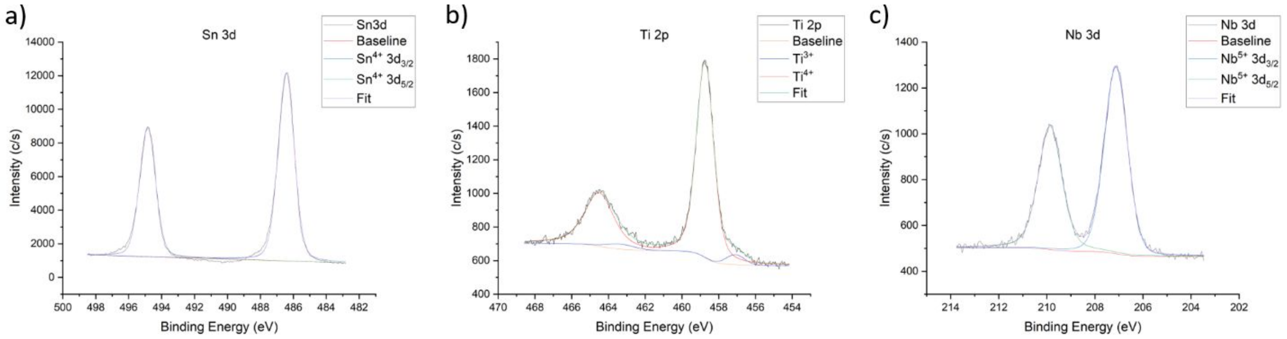

Figure S6. Fits of the high-resolution scans of Sn 3d, Ti 2p and Nb 3d core levels for STN 5 850.

Gas sensors preparation and characterization

An appropriate pneumatic system based on MKS mass flow controllers was used to set gas flows and concentrations of the target gas injected in an hermetically sealed chamber with cylindrical shape (volume: 622 cm^3). Sensor signal baselines were let stabilized at the beginning of each sensing measurement, by keeping the sensors at their working temperature under a continuous flow (500 sccm) of either synthetic dry or wet air. Wet conditions were obtained by fluxing synthetic air through a bubbler filled with deionized water. The chamber was equipped with a gas diffuser at the center, and sensors were positioned circularly around it. The filling time of the chamber, calculated as $\frac{Chamber\ volume}{SCCM} = \frac{622cm^3}{500\frac{cm^3}{min}}$, was about 1 min 15 sec. The chamber hosted up to eight sensors simultaneously, together with a temperature and humidity sensor (Honeywell HIH-4000 humidity). Gases were fluxed from certified bottles with known concentrations. Power suppliers (Aim TTi) and a multimeter (K2000 (Keitheley)) were used to provide the electrical current to the sensor heater and to read the electrical conductance of the sensing film, respectively. The electronic system for the sensing material readout was based on an operational amplifier (OA). Sensor data were collected by using a data acquisition software developed in LabVIEW.

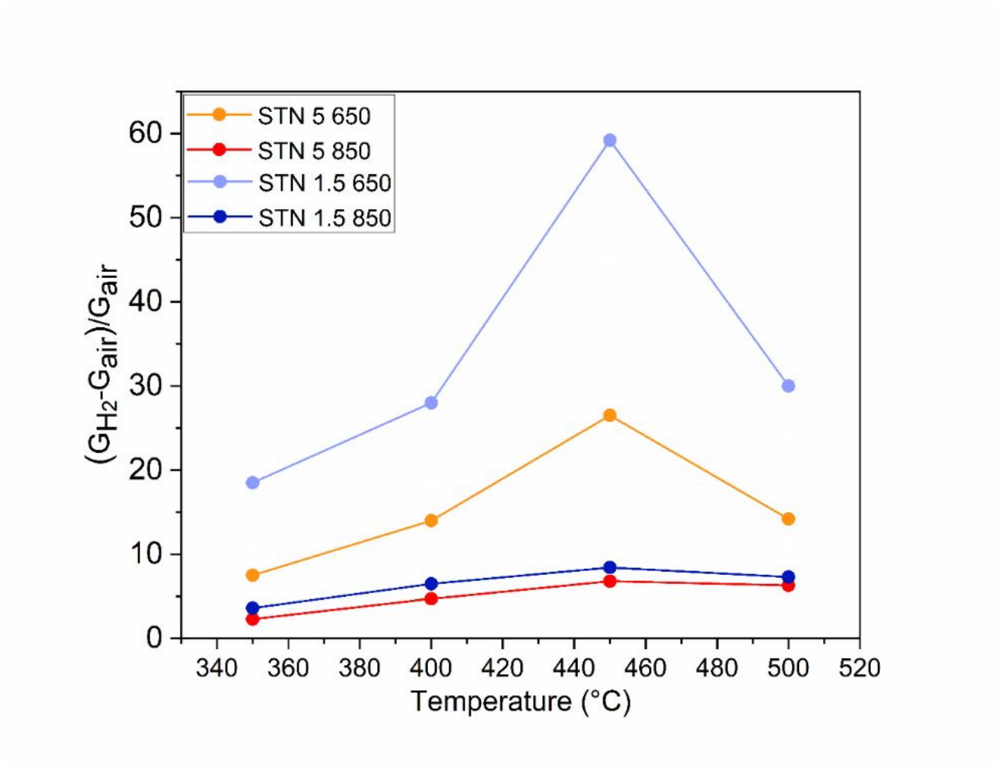

Figure S7. Response of STN films to 50 ppm of H_2 at different working temperatures, at which the STN 650 films reveal a greater dependence than the STN 850 ones.

Table S2. Power law function and linear fit parameters for H_2 calibration curves (see Figure 4.b)

parameter	**STN 5 650**	**STN 5 850**	**STN 1.5 650**	**STN 1.5 850**
power law function $R = ax^b$				
a	7.42 ± 0.75	1.39 ± 0.20	16.67 ± 0.72	1.97 ± 0.18
b	0.33 ± 0.03	0.41 ± 0.04	0.33 ± 0.01	0.38 ± 0.03
linear fit $R = cx$				
c	4.82 ± 0.31	0.91 ± 0.05	12.10 ± 1.05	1.39 ± 0.12

Table S3. Mean response and recovery time for STN as calculated from the graph in **Figure 4c**.

**Response time**				
**STN 5 650**	**STN 5 850**	**STN 1.5 650**	**STN 1.5 850**	**SnO_2 650**
1 min 50 s	1 min 55 s	5 min 10 s	1 min 30 s	5 min 40 s
**Recovery time**				
**STN 5 650**	**STN 5 850**	**STN 1.5 650**	**STN 1.5 850**	**SnO_2 650**
6 min 40 s	7 min 55 s	5 min 50 s	6 min 40 s	5 min 30 s

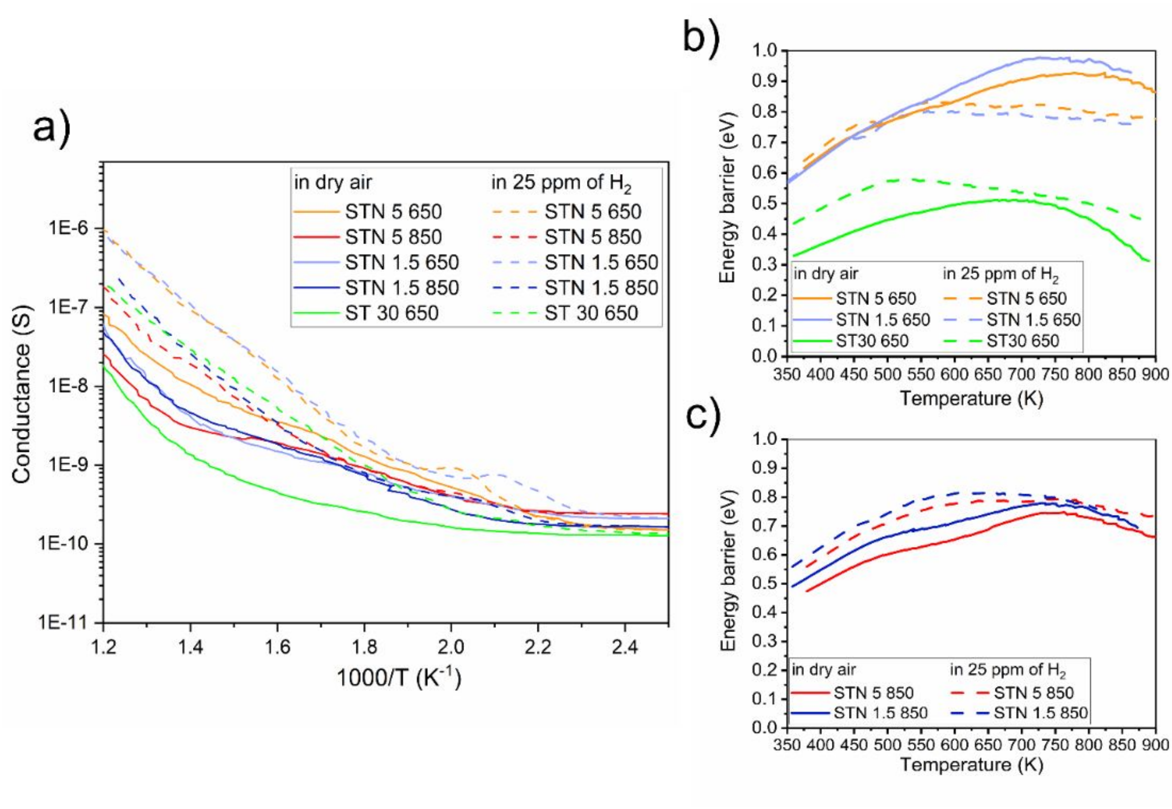

Figure S8. a) Films conductance vs. temperature in dry air (solid line) and 25 ppm of H_2 (dot line). Energy barrier dependence on temperature in dry air (solid line) and 25 ppm of H_2 (dot line) for **b)** STN 650 and **c)** STN 850. The method used for the energy barrier measurements is reported in [15].

References

(1) Speranza, G.; Canteri, R. RxpsG a New Open Project for Photoelectron and Electron Spectroscopy Data Processing. *SoftwareX* **2019**, *10*, 100282. https://doi.org/10.1016/j.softx.2019.100282.

(2) ISO [International Organization for Standardization]. Determination of the Specific Surface Area of Solids by Gas Adsorption - BET Method (ISO 9277:2010(E)). *Ref. number ISO* **2010**, *9277* (9277), 30 pp.

(3) Howard, C. J.; Sabine, T. M.; Dickson, F. Structural and Thermal Parameters for Rutile and Anatase. *Acta Crystallogr. Sect. B Struct. Sci.* **1991**, *47* (4), 462–468. https://doi.org/10.1107/S010876819100335X.

(4) Burdett, J. K.; Hughbanks, T.; Miller, G. J.; Richardson, J. W.; Smith, J. V. Structural-Electronic Relationships in Inorganic Solids: Powder Neutron Diffraction Studies of the Rutile and Anatase Polymorphs of Titanium Dioxide at 15 and 295 K. *J. Am. Chem. Soc.* **1987**, *109* (12), 3639–3646. https://doi.org/10.1021/ja00246a021.

(5) Restori, R.; Schwarzenbach, D.; Schneider, J. R. Charge Density in Rutile, TiO_2. *Acta Crystallogr. Sect. B Struct. Sci.* **1987**, *43* (3), 251–257. https://doi.org/10.1107/S0108768187097921.

(6) SEKI, H.; ISHIZAWA, N.; MIZUTANI, N.; KATO, M. High Temperature Structures of the Rutile-Type Oxides, TiO_2 and SnO_2. *J. Ceram. Assoc. Japan* **1984**, *92* (1064), 219–223. https://doi.org/10.2109/jcersj1950.92.1064_219.

(7) Ballirano, P.; Caminiti, R. Rietveld Refinements on Laboratory Energy Dispersive X-Ray Diffraction (EDXD) Data. *J. Appl. Crystallogr.* **2001**, *34* (6), 757–762. https://doi.org/10.1107/S0021889801014728.

(8) Baur, W. H.; Khan, A. A. Rutile-Type Compounds. IV. SiO_2, GeO_2 and a Comparison with Other Rutile-Type Structures. *Acta Crystallogr. Sect. B Struct. Crystallogr. Cryst. Chem.* **1971**, *27* (11), 2133–2139. https://doi.org/10.1107/S0567740871005466.

(9) Bolzan, A. A.; Fong, C.; Kennedy, B. J.; Howard, C. J. Structural Studies of Rutile-Type Metal Dioxides. *Acta Crystallogr. Sect. B Struct. Sci.* **1997**, *53* (3), 373–380. https://doi.org/10.1107/S0108768197001468.

(10) Cromer, D. T.; Herrington, K. The Structures of Anatase and Rutile. *J. Am. Chem. Soc.* **1955**, *77* (18), 4708–4709. https://doi.org/10.1021/ja01623a004.

(11) Gonschorek, W. X-Ray Charge Density Study of Rutile (TiO_2). *Zeitschrift für Krist.* **1982**, *160* (3–4), 187–203. https://doi.org/10.1524/zkri.1982.160.3-4.187.

(12) Haines, J.; Léger, J. M. X-Ray Diffraction Study of the Phase Transitions and Structural Evolution of Tin Dioxide at High Pressure:FfRelationships between Structure Types and Implications for Other Rutile-Type Dioxides. *Phys. Rev. B* **1997**, *55* (17), 11144–11154. https://doi.org/10.1103/PhysRevB.55.11144.

(13) Henderson, C. M. B.; Knight, K. S.; Lennie, A. R. Temperature Dependence of Rutile (TiO_2) and Geikielite (MgTiO3) Structures Determined Using Neutron Powder Diffraction. *Open Mineral. J.* **2009**, *3* (1), 1–11. https://doi.org/10.2174/1874456700903010001.

(14) Jensen, K. M. Ø.; Christensen, M.; Juhas, P.; Tyrsted, C.; Bøjesen, E. D.; Lock, N.; Billinge, S. J. L.; Iversen, B. B. Revealing the Mechanisms behind SnO_2 Nanoparticle Formation and Growth during Hydrothermal Synthesis: An In Situ Total Scattering Study. *J. Am. Chem. Soc.* **2012**, *134* (15), 6785–6792. https://doi.org/10.1021/ja300978f.

(15) Lantto, V.; Rompplainen, P.; Leppävuori, S. A Study of the Temperature Dependence of the Barrier Energy in Porous Tin Dioxide. *Sensors and Actuators* **1988**, *14* (2), 149–163. https://doi.org/10.1016/0250-6874(88)80062-3.
